# Defective Autophagy in T Cells Impairs the Development of Diet-Induced Hepatic Steatosis and Atherosclerosis

**DOI:** 10.3389/fimmu.2018.02937

**Published:** 2018-12-12

**Authors:** Jacob Amersfoort, Hidde Douna, Frank H. Schaftenaar, Amanda C. Foks, Mara J. Kröner, Peter J. van Santbrink, Gijs H. M. van Puijvelde, Ilze Bot, Johan Kuiper

**Affiliations:** Division of BioTherapeutics, LACDR, Leiden University, Leiden, Netherlands

**Keywords:** autophagy, Atg7, T cells, steatosis, atherosclerosis

## Abstract

Macroautophagy (or autophagy) is a conserved cellular process in which cytoplasmic cargo is targeted for lysosomal degradation. Autophagy is crucial for the functional integrity of different subsets of T cells in various developmental stages. Since atherosclerosis is an inflammatory disease of the vessel wall which is partly characterized by T cell mediated autoimmunity, we investigated how advanced atherosclerotic lesions develop in mice with T cells that lack autophagy-related protein 7 (Atg7), a protein required for functional autophagy. Mice with a T cell-specific knock-out of Atg7 (Lck-Cre Atg7^f/f^) had a diminished naïve CD4^+^ and CD8^+^ T cell compartment in the spleen and mediastinal lymph node as compared to littermate controls (Atg7^f/f^). Lck-Cre Atg7^f/f^ and Atg7^f/f^ mice were injected intravenously with rAAV2/8-D377Y-mPCSK9 and fed a Western-type diet to induce atherosclerosis. While Lck-Cre Atg7^f/f^ mice had equal serum Proprotein Convertase Subtilisin/Kexin type 9 levels as compared to Atg7^f/f^ mice, serum cholesterol levels were significantly diminished in Lck-Cre Atg7^f/f^ mice. Histological analysis of the liver revealed less steatosis, and liver gene expression profiling showed decreased expression of genes associated with hepatic steatosis in Lck-Cre Atg7^f/f^ mice as compared to Atg7^f/f^ mice. The level of hepatic CD4^+^ and CD8^+^ T cells was greatly diminished but both CD4^+^ and CD8^+^ T cells showed a relative increase in their IFNγ and IL-17 production upon Atg7 deficiency. Atg7 deficiency furthermore reduced the hepatic NKT cell population which was decreased to < 0.1% of the lymphocyte population. Interestingly, T cell-specific knock-out of Atg7 decreased the mean atherosclerotic lesion size in the tri-valve area by over 50%. Taken together, T cell-specific deficiency of Atg7 resulted in a decrease in hepatic steatosis and limited inflammatory potency in the (naïve) T cell compartment in peripheral lymphoid tissues, which was associated with a strong reduction in experimental atherosclerosis.

## Introduction

Atherosclerosis is an autoimmune-like disease of the vessel wall in which local accumulation of (modified) lipoproteins elicits an inflammatory response, which is among others T cell-mediated (1). The progression and stability of an atherosclerotic lesion largely depends on the subtype of the T cell, as CD4^+^ T helper cells are generally considered atherogenic, while T regulatory (Treg) cells predominantly act atheroprotective (1). T helper 1 (Th1) cells represent a major fraction of the T cells which drive local inflammation through the secretion of inflammatory cytokines such as interferon-gamma (IFNy) (2, 3). In contrast, Treg cells are immunosuppressive T cells, which can inhibit effector T cells and other immune cells in lymphoid tissues, and atherosclerotic lesions. Treg cells act via direct cell-cell interactions and via secretion of cytokines such as interleukin (IL)-10 and transforming growth factor β (4, 5). Other T helper cell subsets besides Th1 and Treg cells have a less dichotomous contribution to atherosclerosis. Th17 cells represent another subset of T helper cells, which are functionally characterized by the secretion of the interleukin IL-17. Th17 cells are involved in mucosal immunity where these cells help clearance of extracellular pathogens. Interestingly, their contribution to the ongoing inflammatory response in atherosclerotic lesions is context-dependent as Th17 cells have been described to have both atheroprotective as atherogenic functions (6). Cytotoxic CD8^+^ T cells exert their inflammatory function by secreting cytokines, by performing cell-lysis via perforin, or granzyme-B and by inducing cell-death of their target cells through Fas-FasL interactions (7). CD8^+^ T cells might diminish the development of atherosclerotic lesions in early stages of the disease by killing macrophages and other antigen-presenting cells but they actually might promote lesion development by secreting pro-inflammatory cytokines (8). Thus, the contribution of CD8^+^ T cells to atherosclerosis seems context-dependent and remains to be elucidated. One particular process which has gained interest in T cells but has not been studied extensively yet in the context of atherosclerosis is macroautophagy.

Macroautophagy (from henceforth called autophagy) is a well-conserved cellular process in which cytoplasmic cargo is (non-)selectively isolated in double-membrane vesicles called autophagosomes and subsequently transported to lysosomes for lysosomal degradation. Various autophagy-related proteins (Atg) contribute to consecutive phases of the autophagic process (9).

The engulfment of cytoplasmic cargo by autophagosomes is mediated by two ubiquitin-like conjugation systems which are involved in the expansion and closure of the autophagosomal membranes. In the Atg12 conjugation system, Atg12 is activated by the E1 enzyme Atg7 after which Atg12 forms a conjugate with Atg5, and forms a complex with Atg16L (10, 11). In mammalian cells, the Atg12-Atg5-Atg16L is bound to the isolation membrane from which it dissociates after its maturation to an autophagosome (12). The other conjugation system involves microtubule-associated protein 1 light chain 3 (LC3) which is activated by Atg7 and subsequently transferred to Atg3 (13). The Atg12-Atg5-Atg16L complex is required for the adequate conjugation of LC3 to the phospholipid phosphatidylethanolamine (PE) by Atg3 (9, 13). Finally, the LC3-PE conjugate subsequently facilitates the tethering and fusion of the autophagosome membrane (14), thus closing the autophagosome.

Under homeostatic conditions, autophagy is important for the quality control of key organelles for example by degrading and recycling damaged or dysfunctional mitochondria (9). Accordingly, genetic blockade of the Atg5 and Atg7 proteins impact the (functional) stability of CD8^+^ T cells (15), Th1 (16), and regulatory T cells (17).

In CD8^+^ T cells for example, deficiency of Atg5 or Atg7 does not affect clonal expansion, but does impair memory formation and survival, which was also associated with an altered metabolic phenotype in Atg7 deficient T cells (15). Genetic blockade of Atg7 decreases the proliferation of activated naïve CD4^+^ T cells whereas pharmacological blockade of autophagy in differentiated Th1 cells blocks proliferation and IFNγ secretion (16). Treg cell-specific genetic blockade of Atg7 does not affect Treg cell development but severely disrupts their immunosuppressive phenotype under activating conditions. Atg7 deficient Treg cells are apoptotic, lose expression of FoxP3, and gain an inflammatory phenotype characterized by high levels of glycolysis and IFNγ secretion (17).

Thus, genetic or pharmacological inhibition of autophagy modulates the inflammatory phenotype of various CD4^+^ and CD8^+^ T cell subsets albeit in different stages, and through different mechanisms. Given the contribution of the aforementioned T cell subsets to the development of atherosclerosis, their reliance on functional autophagy and the therapeutic implication of certain autophagy inhibitors (such as chloroquine) to treat cardiovascular disease (18), we aimed in this study to determine how atherosclerosis is affected by T cell-specific deletion of Atg7.

Here, we show that atherosclerosis development is severely hampered in mice with T cell-specific Atg7 deficiency and that this is associated with decreased hepatic steatosis and by decreased frequencies of CD4^+^, CD8^+^ T cells, and natural killer T (NKT) cells.

## Materials and Methods

### Mice

All animal work was performed according to the guidelines of the European Parliament Directive 2010/63EU and the experimental work was approved by the Animal Ethics committee of Leiden University. *B6.Cg-Atg7*<*tm1Tchi*> (Atg7^f/f^) and *B6.Cg-Tg(Lck-cre)1Jtak* (Lck-Cre) mice were provided by the RIKEN BRC through the National Bio-Resource Project of the MEXT, Japan. To generate mice with T cell-specific deficiency of Atg7, Atg7^f/f^ mice were crossed with mice expressing Cre recombinase under control of the *Lck* promotor (Lck-Cre), thus creating Lck-Cre Atg7^f/f^ mice. Atg7^f/f^ littermates served as controls. 18 week old Lck-Cre Atg7^f/f^ mice and their littermates were used to examine the effects of Atg7 deficiency on the T cell populations in the blood, spleen, and mediastinal lymph nodes (medLN) under normolipidemic conditions.

### Flow Cytometry

Spleens and mediastinal lymph nodes (medLN) were isolated and mashed through a 70 μm cell strainer. Erythrocytes were subsequently eliminated from the blood and spleen by incubating the cells with ACK erythrocyte lysis buffer to generate a single-cell suspension prior to staining of surface markers. To isolate hepatic lymphocytes, non-parenchymal cells from the liver were first separated from parenchymal cells by centrifugation at low speed. Subsequently, the non-parenchymal cells were put on a Lympholyte gradient (Cedarlane) to isolate hepatic lymphocytes prior to staining of surface markers. For analysis of surface markers identifying CD4^+^, CD8^+^, and NKT cells, splenocytes, or lymphocytes were stained at 4°C for 30 min. in staining buffer [phosphate buffered saline with 2% (vol/vol) fetal bovine serum (FBS)]. All antibodies used for staining of surface markers or transcription factors were from Thermo Fischer and BD Biosciences (Supplementary Table 1). To identify NKT cells, an allophycocyanin labeled α-GalCer/CD1d tetramer kindly provided by the NIH tetramer core facility (Atlanta, GA) was used.

For staining of intracellular cytokines, splenocytes, or liver-derived lymphocytes were incubated for 4 h with 50 ng/mL phorbol myristate acetate (PMA) (Sigma), 500 ng/mL ionomycin (Sigma), and Brefeldin A (ThermoFisher). Extracellular staining was then performed with subsequent fixation and permeabilization with Cytofix/Perm and Perm Wash buffer (both from BD Biosciences). Staining for intracellular cytokines was performed in Perm Wash Buffer after which the cells were washed with staining buffer prior to flow cytometric analysis.

Flow cytometric analysis was performed on a FACSCantoII (BD Biosciences) and data was analyzed using Flowjo software (TreeStar).

### T Cell Proliferation

Splenocytes were isolated from Lck-Cre Atg7^f/f^ or Atg7^f/f^ mice and activated with anti-CD3e (1 μg/mL) and anti-CD28 (0.5 μg/mL) (both from ThermoFischer) for 72 h and incubated with 0.5 μCi/well ^3^H-thymidine (Perkin Elmer, The Netherlands) for the last 16 h. The amount of ^3^H-thymidine incorporation was measured using a liquid scintillation analyzer (Tri-Carb 2900R). Responses are expressed as the mean disintegrations per minute (dpm). The stimulation index (s.i.) was defined by dividing the dpm under activated conditions by the dpm under non-activated conditions per mouse.

### Atherosclerosis

To investigate atherosclerosis in Lck-Cre Atg7^f/f^ and Atg7^f/f^ mice, 18 to 20-week old female mice were administered rAAV2/8-D377Y-mPCSK9 (5 × 10^11^genome copies/mouse) by i.v. injection (19), which results in overexpression of PCSK9 and subsequent development of atherosclerosis. After 1 day, mice were switched from a normal chow diet to a Western-type Diet (WTD, Special Diet Services) containing 0.25% cholesterol and 15% cocoa butter. The weight of the mice was monitored regularly. After 22 weeks, the mice were anesthetized by subcutaneous injections with ketamine (100 mg/mL), sedazine (25 mg/mL), and atropine (0.5 mg/mL) after which their vascular system was perfused with PBS at a continuous low flow via heart puncture in the left ventricle. Next, the spleen, liver, and inguinal white adipose tissue (iWAT) were collected for further processing. The hearts were collected, embedded in O.C.T. compound (Sakura), and then snap-frozen using dry-ice and stored at −80°C until further use.

### Histology

To examine atherosclerotic lesions in the aortic root, the hearts were sectioned horizontally to the aortic axis, and toward the aortic arch. Upon identification of the aortic root, defined by the trivalve leaflets, 10 μm sections were collected. In order to visualize hepatic steatosis, a small piece of liver was dissected upon sacrifice, and fixed using Zinc Formal-Fixx solution. Subsequently, the livers were embedded in O.C.T. compound after which 8 μm sections were prepared. After fixation with Zinc Formal-Fixx solution (Thermo Fischer) the neutral lipids in both aortic root and liver were stained using Oil-red-O (Sigma). Collagen content of the plaques was stained using a Mason's Trichrome staining kit (Sigma). Monocytes and macrophages were detected using a Moma2 primary antibody (Serotec) and biotinylated secondary antibody and visualized using the VECTASTAIN^®^ Avidin-Biotin Complex Staining Kit (Vector Labs). After dissection of the iWAT, the tissue was fixed, dehydrated, and subsequently embedded in paraffine and sectioned in 8 μm sections. After this, iWAT sections were deparaffinized and rehydrated prior to staining with Gill No. 3 hematoxylin solution (Sigma). Adipocyte size was quantified using the Adiposoft plugin in Fiji software (20). The liver sections were examined visually to assess the degree of Oil-red-O staining as a measure for hepatic steatosis. For morphologic and morphometric analysis of the aortic root, the slides were analyzed using a Leica DM-RE microscope, and LeicaQwin software (Leica Imaging Systems). Mean plaque size (in μm^2^) was calculated from five sequential sections, displaying the highest plaque content. (Immuno)histochemical stainings were expressed as the percentage of positive stained area of the total lesion area. All morphometric analyses were performed by blinded independent operators.

### Confocal Microscopy

Fresh frozen livers of Atg7^f/f^ and Lck-Cre Atg7^f/f^ mice were sectioned on a cryostat in 8 μm thick sections. Sections were fixed in an ice-cold 75% acetone/25% ethanol mix and blocked in 2% normal goat serum and 3% bovine serum albumin (Sigma). Next, the sections were incubated overnight at 4°C with antibodies detecting CD68 and CD3e (both from Abcam). Subsequently, the sections were washed and incubated with antibodies conjugated to Alexa Fluor 488 or Alexa Fluor 647 (both from Abcam). Nuclei were visualized using Fluoroshield mounting medium with DAPI (Sigma). T cells were manually quantified while blinded to the genotype using Fiji software. Three images were analyzed of every liver section and three liver sections were analyzed per mouse. Confocal images were acquired using a 20x objective on a Nikon TiE 2000 confocal microscope.

### Real-Time Quantitative PCR

RNA was extracted from mechanically disrupted livers by using Trizol reagent per the manufacturer's instructions (Invitrogen) after which cDNA was generated using RevertAid M-MuLV reverse transcriptase according to the manufacturer's protocol (Thermo Scientific). Quantitative gene expression analysis was performed using Power SYBR Green Master Mix on a 7500 Fast Real-Time PCR system (Applied Biosystems). Gene expression was normalized to housekeeping genes (Supplementary Table 2).

### Immunoblot

Immunoblot was performed as described previously (21) with minor modifications. Briefly, CD4^+^ T cells were isolated using MACS microbeads (Miltenyi Biotec). For protein isolation, cells were lysed with 1xRIPA (Cell Signaling Technology) supplemented with cOmplete™ Protease Inhibitor Cocktail (Sigma) and 0.1% SDS for 30 min on ice. Proteins were detected using rabbit-anti-mouse Atg7 (Abcam) and rabbit-anti-mouse β-actin (Novus Biologicals) antibodies and visualized using chemiluminescence.

### Serum Analysis

The serum PCSK9 concentrations were determined using the Mouse Proprotein Convertase 9 DuoSet ELISA kit (R&D Systems) per the manufacturer's instructions. Concentration of total cholesterol in the serum was determined by an enzymatic colorimetric assay (Roche Dagnostics). Precipath (standardized serum, Roche Diagnostics) was used as an internal standard in the measurements for cholesterol.

### Statistical Analysis

For statistical analysis, a two-tailed Student's *T*-test was used to compare individual groups with Gaussian distributed data. Non-parametric data was analyzed using a Mann-Whitney *U*-test. Data from three or more groups were analyzed using a one-way ANOVA whereas data from three groups with more than one variable were analyzed by a two-way ANOVA, both with a subsequent Sidak multiple comparison test. Correlation was assessed using Pearson's correlation coefficient. A *p*-value below 0.05 was considered significant. Throughout the manuscript a ^*^indicates *p* < 0.05, ^**^indicates *p* < 0.01, ^***^indicates *p* < 0.001, and ^****^indicates *p* < 0.0001.

## Results

### Atg7 Deficiency Affects T Cell Populations in medLN and Spleen

**Figure 1:**
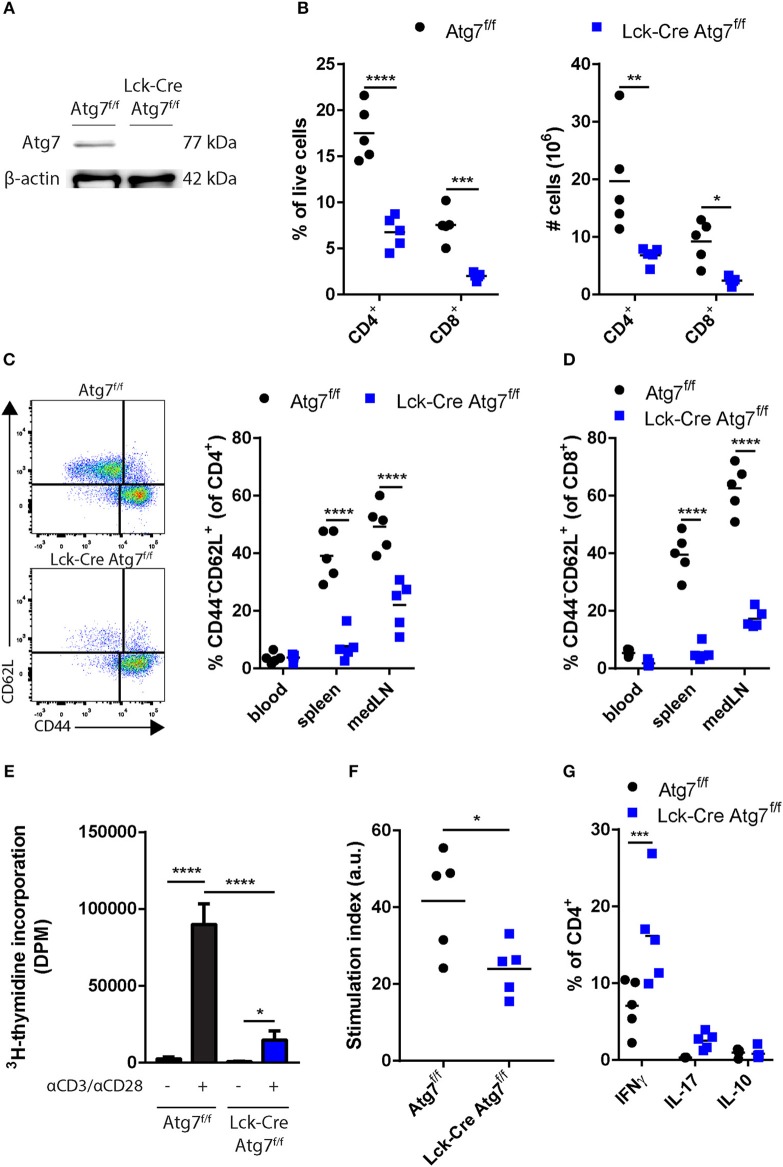
Effect of Atg7 deficiency on CD4^+^ and CD8^+^ T cells. **(A)** Immunoblot of Atg7 in CD4^+^ T cells. β-actin was used as a loading control. **(B)** Percentage and numbers of CD4^+^ and CD8^+^ cells in the live lymphocyte fraction of spleens of indicated genotypes. **(C)** Gating and percentages of naive T cells in the CD4^+^ compartment. **(D)** Percentages of naive T cells in the CD8^+^ compartment. **(E)** T cell proliferation in stimulated or unstimulated splenocyte cultures. Expressed as mean ± standard deviation. **(F)** Stimulation index as calculated by dividing DPM of anti-CD3/anti-CD28 stimulated splenocytes by the DPM of non-stimulated splenocytes for each genotype. **(G)** Quantification of cytokine producing CD4^+^ T cells. ^*^p < 0.05, ^**^p < 0.01, ^***^p < 0.001, ^****^p < 0.0001.

To confirm that Cre recombinase expression in the Lck-Cre Atg7^f/f^ mice was sufficiently high to induce Atg7 deficiency in T cells, we isolated splenic CD4^+^ T cells from Lck-Cre Atg7^f/f^ mice and Atg7^f/f^ littermates, and analyzed Atg7 expression on a protein level by immunoblot. Atg7 was successfully knocked out as Atg7 could not be detected by immunoblot in CD4^+^ T cells from Lck-Cre Atg7^f/f^ mice whereas the control showed a clear Atg7 signal (Figure 1A). As described previously, T cell-specific deficiency of Atg7 compromises single positive T cell generation in the thymus and induces peripheral lymphopenia (22). We identified CD4^+^ and CD8^+^ T cells from peripheral tissues by flow cytometry using the gating strategy described in Figure S1A. The spleens of Lck-Cre Atg7^f/f^ mice indeed contained significantly fewer CD4^+^ and CD8^+^ cells both in percentage and numbers (Figure 1B). We assessed the naïve CD4^+^ T cell compartment in blood, spleen, and mediastinal lymph node (medLN, the lymph node draining from the trivalve area) as CD4^+^ T cells can be activated by lipoprotein-derived antigens during atherosclerosis (3). The percentage of CD4^+^ naïve T (Tn) cells as defined by CD4^+^CD44^−^CD62L^+^ was significantly decreased in the spleen and medLN of Lck-Cre Atg7^f/f^ mice as compared to Atg7^f/f^ mice (Figure 1C). Accordingly, the number of CD4^+^ Tn cells was decreased in the spleen of Lck-Cre Atg7^f/f^ mice as compared to Atg7^f/f^ mice (Figure S1B). Similar to the percentage of CD4^+^ Tn cells, the percentage of CD8^+^ Tn cells was decreased in the same tissues in Lck-Cre Atg7^f/f^ mice (Figure 1D) and the number of CD8^+^ Tn cells in the spleen was decreased as well in Lck-Cre Atg7^f/f^ mice (Figure S1C). Next, we assessed whether the proliferative capacity of Atg7 deficient T cells is impaired which was confirmed using a proliferation assay measuring ^3^H-thymidine incorporation with/without anti-CD3 and anti-CD28 stimulation (Figure 1E). The stimulation index was calculated and this parameter showed the proliferation of Atg7 deficient T cells was lower than Atg7 competent T cells (Figure 1F). The percentage of IFNγ, IL-17, and IL-10 was measured in splenic CD4^+^ T cells using flow cytometry to assess the inflammatory capacity of the diminished T cell population in Lck-Cre Atg7^f/f^ mice. Atg7 deficiency significantly increased the percentage of IFNγ-producing CD4^+^ T cells (Figure 1G), causing the absolute number of IFNγ producing CD4^+^ T cells to be unaltered between both genotypes (Figure S1D). The percentages of IL-17 and IL-10 producing T cells were unaltered.

Together, these results indicate that the generation of mice with Atg7 deficient T cells was successful from a genotypic and phenotypic perspective.

### Atg7 Deficiency in T Cells Decreased Hepatic Steatosis and Dyslipidemia During WTD-Induced Atherosclerosis

**Figure 2:**
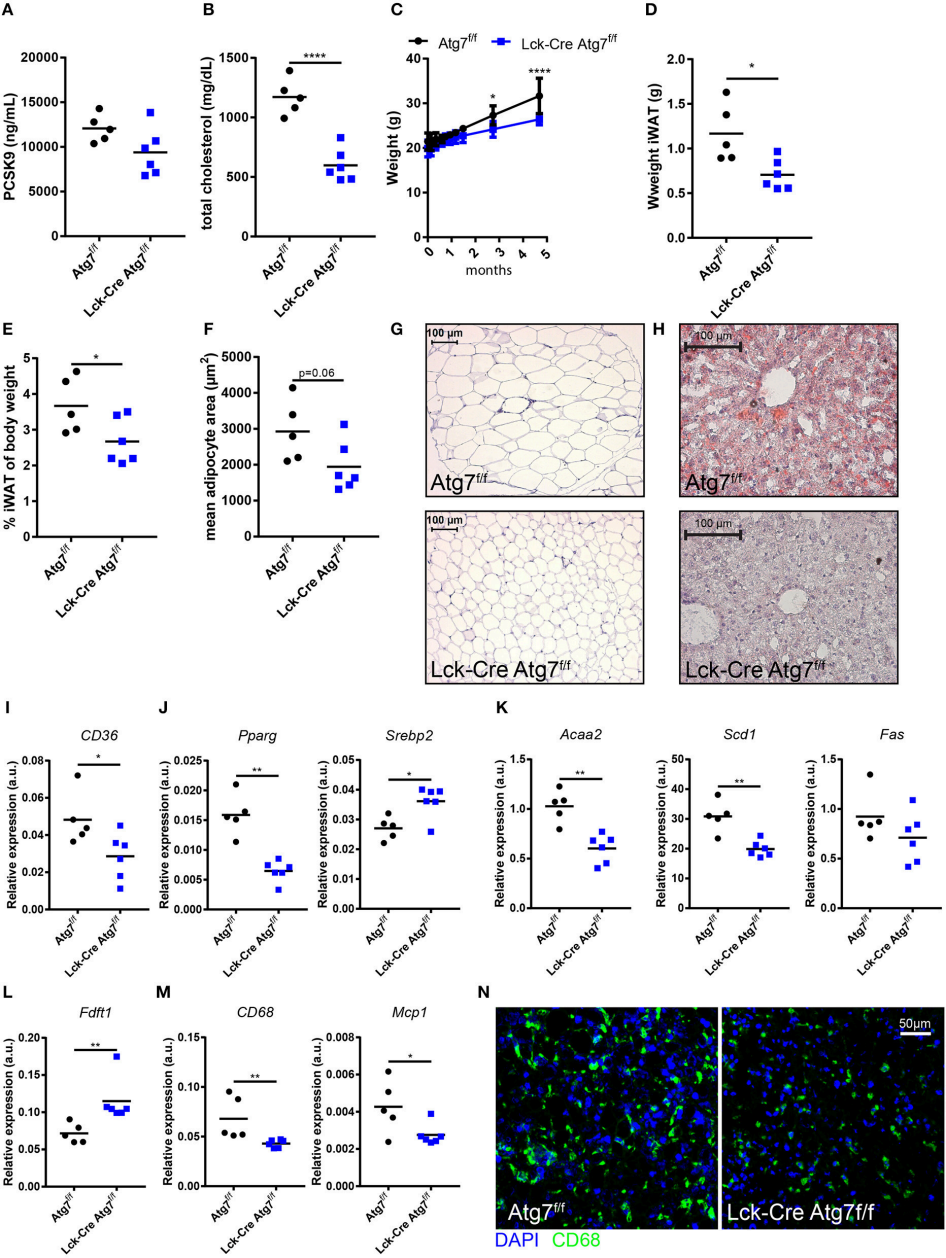
Atg7 deficiency in T cells decreased hepatic steatosis and dyslipidemia. **(A)** PCSK9 levels in serum. **(B)** Total cholesterol levels in serum. **(C)** Weight of the mice over the course of the experiment. **(D)** Weight inguinal white adipose tissue. (iWAT). **(E)** Weight iWAT as a percentage of body weight at sacrifice. **(F)** Quantification of adipocyte area in iWAT. **(G)** Representative sections of iWAT used for adipocyte size quantification in **(F)**. **(H)** Representative Oil-Red-O stained sections of liver. **(I)** Gene expression in liver of scavenger receptor CD36. **(J)** Gene expression in liver of transcription factors Pparg and Srebp2. **(K)** Gene expression in liver of fatty acid synthesis genes Acaa2, Scdl, and Fas. **(L)** Gene expression in liver of enzyme involved in cholesterol synthesis, Fdftl. **(M)** Gene expression in liver of macrophage lineage marker CD68 and monocyte chemoattractant Mcp 1. **(N)** Representative confocal images of CD68 expression in liver sections. ^*^p < 0.05, ^**^p < 0.01, ^****^p < 0.0001.

As we were interested in the impact of Atg7 deficiency in T cells on the development of diet-induced experimental atherosclerosis, we injected Lck-Cre Atg7^f/f^ and Atg7^f/f^ mice with an adenoassociated virus encoding an active variant of PCSK9 (rAAV2/8-D377Y-mPCSK9) and fed them a WTD for 22 weeks to induce advanced atherosclerosis. As a result of overexpression of murine PCSK9 in the liver, the LDL receptor is targeted for lysosomal degradation and upon WTD-feeding circulating cholesterol levels are significantly elevated, to a level which is comparable to WTD-fed LDL receptor deficient mice (19). After 4 weeks of WTD, the levels of mPCSK9, and cholesterol in the serum were measured to confirm that the viral transduction was successful. In general, the levels of serum PCSK9 exceeded 10,000 ng/mL which is sufficiently high to induce atherosclerosis upon prolonged WTD feeding (19). Moreover, the levels of serum PCSK9 did not differ between both genotypes (Figure 2A). Interestingly, despite the serum PCSK9 levels being equal between both genotypes, serum cholesterol was significantly lower after 4 weeks of WTD in Lck-Cre Atg7^f/f^ mice as compared to Atg7^f/f^ mice (Figure 2B). The lowest serum PCSK9 levels in Lck-Cre Atg7^f/f^ (~8,000 ng/mL) in this study were sufficiently high to be associated with serum cholesterol levels comparable to what we observed in Atg7^f/f^ mice (19), suggesting that Atg7 deficiency results in decreased serum cholesterol levels after WTD feeding. Furthermore, after prolonged WTD feeding, the weight of Lck-Cre Atg7^f/f^ mice was lower compared to Atg7^f/f^ mice (Figure 2C) and in line, the weight of the iWAT was lower in Lck-Cre Atg7^f/f^ mice (Figure 2D) also when this was corrected for body weight at sacrifice (Figure 2E). Accordingly, the mean adipocyte size was decreased in Lck-Cre Atg7^f/f^ mice as compared to Atg7^f/f^ mice, although this did not reach significance (*p* = 0.06, Figures 2F,G). As compared to Atg7^f/f^ mice, the livers of Lck-Cre Atg7^f/f^ mice appeared less steatotic (Figure 2H), which is consistent with a decrease in total serum cholesterol levels. Next, we measured the expression of genes that are associated with hepatic steatosis (23) and the expression of a number of additional genes involved in lipid metabolism was decreased in livers of Lck-Cre Atg7^f/f^ mice. The expression of CD36, for example, a scavenger receptor known to mediate the uptake of native and modified lipoproteins, was decreased in Lck-Cre Atg7^f/f^ mice (Figure 2I). Furthermore, the expression of the transcription factor peroxisome proliferator activated receptor gamma (*Pparg*), which is known to be associated with hepatic steatosis (24), was decreased while the expression of sterol regulatory element binding protein 2 (*Srebp2*) was increased in the liver of Lck-Cre Atg7^f/f^ mice (Figure 2J). On the other hand, the mRNA expression of genes involved in fatty acid synthesis in the liver was decreased, including *Acaa2, Scd1*, and *Fas* (Figure 2K). In line with *Srebp2* expression being increased, the expression of *Fdft1*, which is involved in cholesterol synthesis, was elevated in Lck-Cre Atg7^f/f^ mice (Figure 2L). The macrophage content in livers of Lck-Cre Atg7^f/f^ mice was also decreased as suggested by the decreased mRNA expression of the macrophage lineage marker *CD68* and the chemokine *Mcp1* (Figure 2M). The decreased CD68 expression in livers of Lck-Cre Atg7^f/f^ mice was confirmed on protein level in confocal images (Figure 2N).

Altogether, T cell-specific Atg7 deficiency hampered WTD-induced hepatic steatosis and dyslipidemia despite successful viral transduction with rAAV2/8-D377Y-mPCSK9.

### Atg7 Deficiency in T Cells Decreases T Cell Abundance in the Liver but Increases Inflammatory Cytokine Production

**Figure 3:**
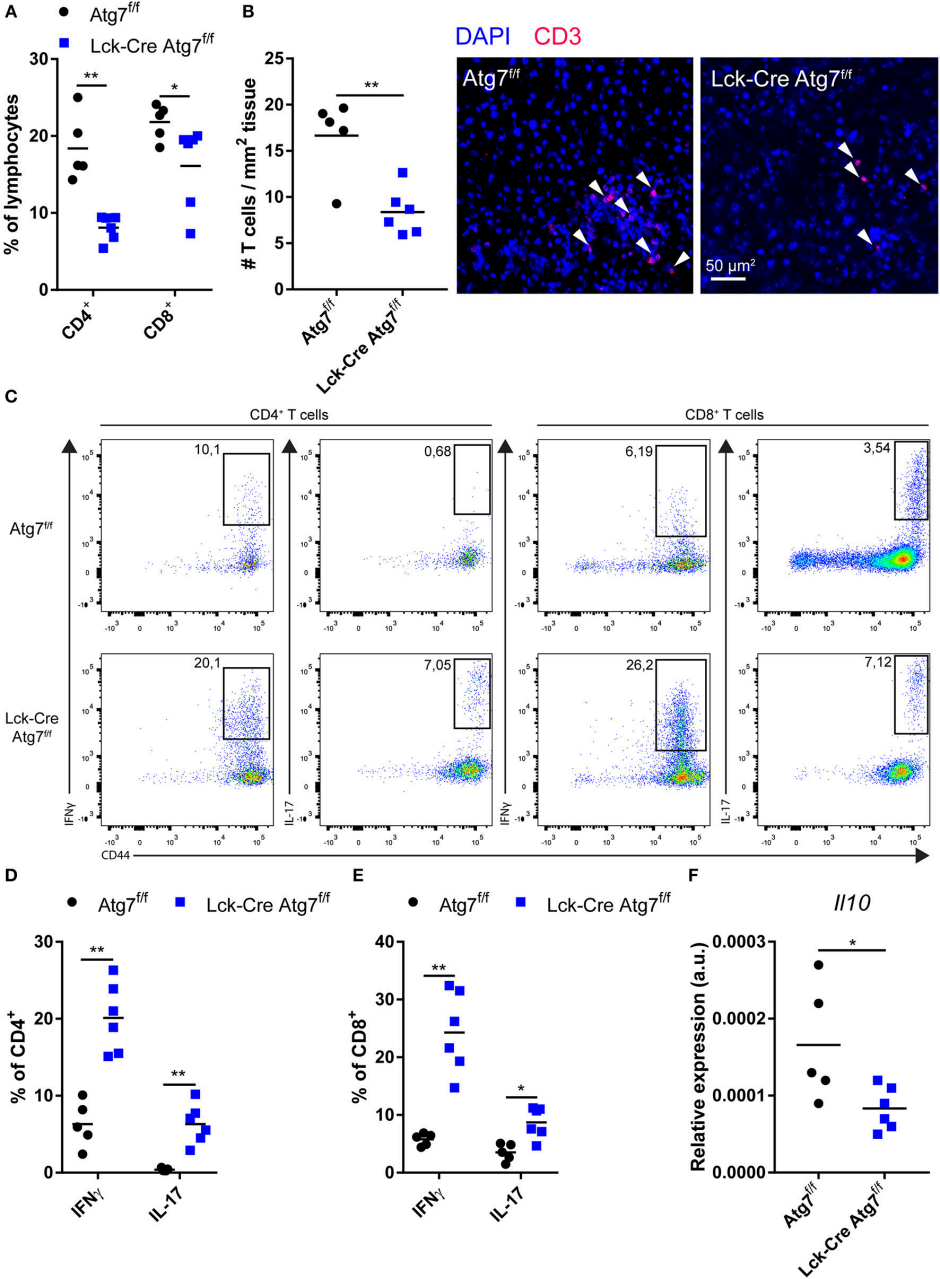
Atg7 deficiency in T cells diminishes T cells in liver but increases relative inflammatory cytokine production. **(A)** Percentage of CD4^+^ and CD8^+^ T cells in live hepatic lymphocyte fraction. **(B)** Quantification and representative confocal images of T cells in liver **(C)** Gating strategy to identify IFNγ and IL-17 producing T cells in liver. **(D)** Percentage of IFNγ and IL-17 producing hepatic CD4^+^ T cells. **(E)** Percentage of iFNγ and IL-17 producing hepatic CD8^+^ T cells. **(F)** *Il10* expression in liver. ^*^p < 0.05, ^**^p < 0.01.

As inflammation is one of the drivers of hepatic steatosis (25), we postulated that Lck-Cre Atg7^f/f^ mice developed less severe hepatic steatosis during our experiments as the inflammatory capacity of the hepatic T cell population was impaired. Therefore, we characterized the CD4^+^ and CD8^+^ T cell populations in the liver. In line with the observations in the spleen, the percentage of CD4^+^ T cells in the lymphocyte fraction of the liver was decreased as a result of Atg7 deficiency (Figure 3A). Additionally, the percentage of CD8^+^ T cells was decreased as well, albeit to a lesser extent (Figure 3A). Quantification of the number of T cells per mm^2^ liver tissue using confocal microscopy showed that the absolute number of T cells was decreased in livers of Lck-Cre Atg7^f/f^ mice as compared to Atg7^f/f^ mice (Figure 3B). We measured the inflammatory capacity of CD4^+^ and CD8^+^ T cells through flow cytometry by measuring the percentage of IFNγ and IL-17 producing cells in both genotypes (Figure 3C). The percentage of IFNγ^+^ cells in the hepatic CD4^+^ T cells showed a 2-fold increase in Atg7 deficient CD4^+^ T cells whereas the percentage of IL-17 producing cells was also increased by Atg7 deficiency going from ~0.4% in Atg7 wildtype CD4^+^ T cells to ~6.3% in Atg7 deficient CD4^+^ T cells (Figure 3D). Similarly, the IFNγ and IL-17 production was increased in Atg7 deficient CD8^+^ T cell compartment as compared to Atg7 wildtype CD8^+^ T cells (Figure 3E). However, this did not result in increased mRNA levels of IFNγ or IL-17 (not detectable) in liver homogenate (Figure S2A). To confirm that the total amount of IFNγ producing T cells was not increased as a result of Atg7 deficiency, we isolated hepatic lymphocytes from normocholesterolemic Atg7^f/f^ and Lck-Cre Atg7^f/f^ mice and quantified the total number of activated IFNγ producing cells. The total number of IFNγ producing lymphocytes was unaltered between Atg7^f/f^ and Lck-Cre Atg7^f/f^ mice (Figure S2B), suggesting that Atg7 deficiency in T cells causes an increase in the percentage of hepatic IFNγ producing T cells but not in the total number of IFNγ producing cells prior to steatosis development. Despite a previous report describing Atg7 deficient Treg cells to express more IFNγ than their Atg7 competent counterparts (17) barely any IFNγ producing T cell in livers of normocholesterolemic mice are FoxP3^+^ (Figure S2C). As the results described above suggested Atg7 deficiency induced the skewing of the diminished T cell population toward an inflammatory phenotype and IL-10 is an anti-inflammatory cytokine, we measured *Il10* expression in the livers of Lck-Cre Atg7^f/f^ and Atg7^f/f^ mice. In line with an inflammatory phenotype and Atg7 deficiency disrupting Treg cell function and stability, the expression of *Il10* was decreased in livers of Lck-Cre Atg7^f/f^ mice as compared to Atg7^f/f^ control mice (Figure 3F). Whether this decrease in *Il10* expression is truly T cell dependent or whether it is due to the less advanced stage of hepatic steatosis in Lck-Cre Atg7^f/f^ mice remains to be determined.

In conclusion, although Atg7 deficiency resulted in a relative increase in T cells which produce inflammatory cytokines, the decrease in CD4^+^ and CD8^+^ T cells in the liver likely impairs the development of hepatic steatosis in Lck-Cre Atg7^f/f^ mice.

### Lack of NKT Cells in Mice With T Cell-Specific Atg7 Deficiency

**Figure 4:**
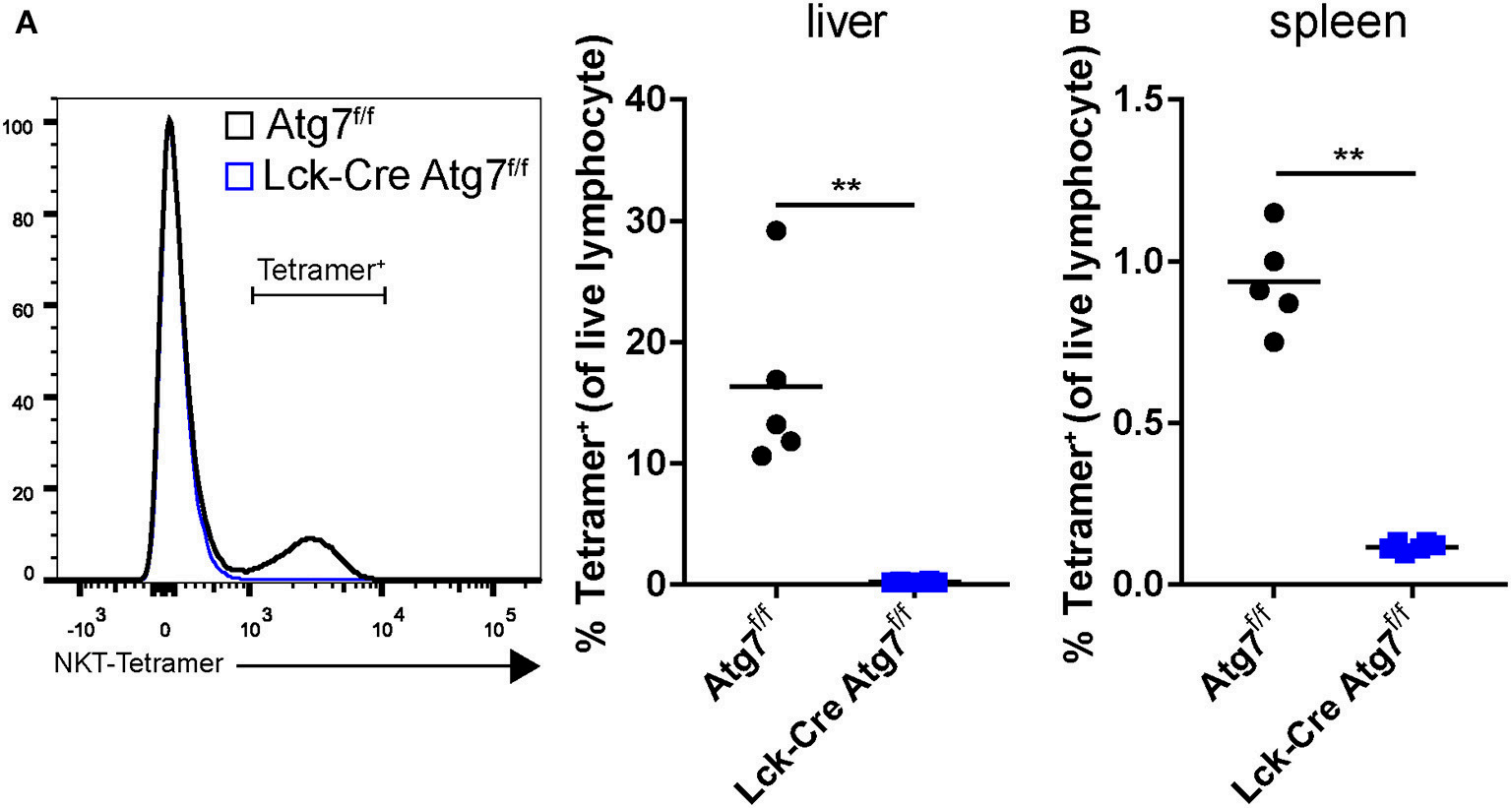
Atg7 deficiency in T cells reduces the amount of NKT cells in liver and spleen. **(A)** NKT cells as detected by an α-GalCer/CDld tetramer staining and presented as a percentage of the lymphocyte fraction in the liver. **(B)** Percentage ofNKT cells in spleen. ^**^p < 0.01.

In mice, natural killer T (NKT) cells represent a relatively large fraction of hepatic lymphocytes (up to 35%). NKT cells recognize lipid-derived antigens when presented on the major-histocompatibility complex-resembling protein CD1d. The most common type of NKT cells in mice is the type I NKT cells, also called invariant NKT cells. Upon stimulation, NKT cells secrete a plethora of cytokines, including Th1-like (IFNγ, TNFα) cytokines and IL-10. As NKT cells have a functional TCRαβ and express one of its proximal signaling kinases Lck (26), we hypothesized that Atg7 deficiency disrupted NKT cell function. Using flow cytometry, we observed that the percentage of NKT cells was severely diminished in the hepatic lymphocyte fractions of Lck-Cre Atg7^f/f^ mice from ~18% to ~0.1% (Figure 4A). Similarly, in the spleens of Lck-Cre Atg7^f/f^, only ~0.1% of the lymphocyte population consisted of NKT cells compared to ~1% in the control (Figure 4B).

Thus, T cell-specific Atg7 deficiency not only diminished the CD4^+^ and CD8^+^ T cell populations but also severely reduced the percentage of hepatic NKT cells, which may have contributed to impaired hepatic steatosis development as suggested by literature (25).

### T Cell Specific Atg7 Deficiency Decreases Atherosclerosis

**Figure 5:**
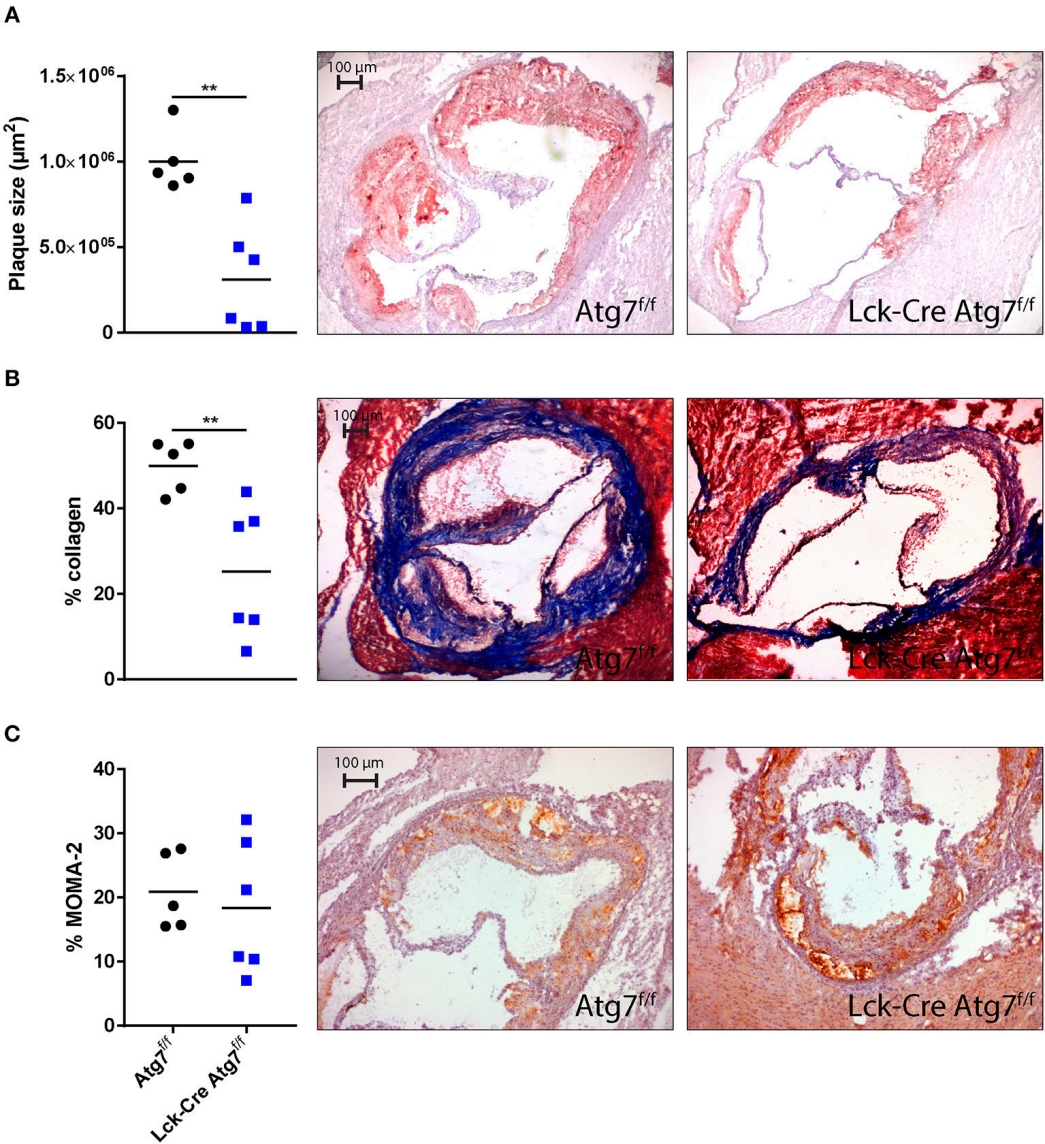
Histological analysis of atherosclerosis in the aortic root. **(A)** Quantification of mean plaque size using Oil-red-O staining. **(B)** Quantification of collagen content using a Masson's Trichrome staining. Collagen fibers are indicated in blue. **(C)** Quantification of monocyte-macrophage content using a MOMA-2 antibody. ^**^p < 0.01.

Since we were interested in the effect of T cell-specific Atg7 deficiency on the development of diet-induced advanced atherosclerosis we quantified atherosclerotic lesion size in the aortic root after 22 weeks of WTD. Lck-Cre Atg7^f/f^ mice developed 50% smaller lesions than Atg7^f/f^ control mice (Figure 5A, *p* < 0.01). Interestingly, the correlation between lesion size and serum total cholesterol levels was stronger in Lck-Cre Atg7^f/f^ mice as compared to Atg7^f/f^ mice (Figures S3A,B), suggesting that T cell-specific Atg7 deficiency renders serum cholesterol to be a stronger driver of atherogenesis in Lck-Cre Atg7^f/f^ mice. Additionally, T cell-specific Atg7 deficiency reduced the collagen content by ~50% (Figure 5B). Lastly, the relative amount of monocytes and macrophages in the lesions was quantified using a MOMA-2 staining. No differences were observed in terms of macrophage content between Lck-Cre Atg7^f/f^ and Atg7^f/f^ mice (Figure 5C).

## Discussion

Different T cell subsets which are crucially involved in the development of atherosclerosis depend on autophagy for their functional integrity and survival. Systemic administration of autophagy inhibitors such as chloroquine or hydroxychloroquine has therapeutic potential to treat atherosclerosis as it shows anti-inflammatory effects in other autoimmune diseases such as rheumatoid arthritis. In mice, systemic low-dose administration of chloroquine inhibits diet-induced atherosclerosis in ApoE-deficient mice (27). To gain more insight in the T cell-specific contribution of the anti-inflammatory effect of autophagy blockade, we genetically blocked autophagy in T cells and studied the impact on diet-induced atherosclerosis in experimental models of disease.

In this model, knock-out of Atg7 in T cells significantly decreased the percentage and numbers of CD4^+^ and CD8^+^ T cells. The percentage of naïve T cells was also decreased in lymphoid tissues, the mediastinal lymph nodes and spleen, in which naïve T cells respond to lesion and lipoprotein-derived antigens. These findings are in line with data that naïve T cells go into apoptosis without functional autophagy (28), which is highly relevant for atherosclerosis research as this would result in relatively fewer T cells to respond to atherosclerosis derived antigens and thus to the ongoing inflammation in atherosclerotic lesions. Atg7 deficiency in T cells impaired their proliferative capacity in a splenocyte culture under anti-CD3 and anti-CD28 antibody induced stimulation, which is in line with a previous report describing that Atg7 deficient naïve CD4^+^ T cells proliferate less after antibody-mediated TCR stimulation (16). TCR stimulation also activated CD8^+^ T cells in the splenocyte culture but as autophagy is not induced upon activation of CD8^+^ T cells (15) and their proliferative capacity is not affected by Atg5 or Atg7 deficiency (15) it is unlikely that the decrease in T cell proliferation we observed in our experiments was CD8^+^ T cell-mediated. Under normolipidemic conditions, the spleens of Lck-Cre Atg7^f/f^ mice contained fewer CD4^+^ T cells although a relatively higher percentage of these CD4^+^ cells secreted IFNγ. Although we did not observe a difference in total numbers of CD4^+^ IFNγ^+^ T cells between Lck-Cre Atg7^f/f^ and Atg7^f/f^ mice, Atg7 deficient T cells have been described to secrete lower amounts of IFNγ and IL-10 and also lower amounts of other T helper cell cytokines including IL-4 and IL-17 (29). These results suggested that Atg7 deficiency severely compromised the inflammatory capacity of all the T helper cell populations in the medLN and spleen in our studies. Therefore, the fact that Atg7 deficient CD4^+^ and CD8^+^ T cells had a higher percentage of IFNγ producing cells than their Atg7 competent counterparts is negated by the diminished number of CD4^+^ and CD8^+^ T cells and their capacity to not only produce but also secrete cytokines in Lck-Cre Atg7^f/f^ mice as compared to Atg7^f/f^ mice.

As LDL receptor competent mice barely develop atherosclerosis we injected Atg7^f/f^ and Lck-Cre Atg7^f/f^ mice with a single injection of rAAV2/8-D377Y-mPCSK9. Compared to Atg7^f/f^ mice, Lck-Cre Atg7^f/f^ mice had lower serum cholesterol levels and less hepatic steatosis under dyslipidemic conditions based on histological evaluation and gene expression of genes associated with hepatic steatosis. As Lck-Cre Atg7^f/f^ mice gained less weight, and had a lower iWAT weight, the decreased extent of hepatic steatosis in these mice had effects beyond just the decrease in circulating cholesterol levels. However, given the role of T cells in the development of obesity-associated inflammation of WAT (30–32), the impaired weight gain could also be explained by impairments in T cell-mediated inflammation of WATs. This indicates that, under dyslipidemia conditions, Atg7 deficiency has impact on the immunometabolic phenotype of mice on a systemic level which is relevant for further research examining autophagy blockade in models of dyslipidemia *in vivo*.

Since hepatic steatosis was decreased upon T cell-specific deletion of Atg7, we hypothesized that this was due to a reduction in the numbers and profile of cytokine secretion of CD4^+^ and CD8^+^ T cells in the livers of Lck-Cre Atg7^f/f^ mice. Similar to the spleens and medLNs during normolipidemia, the livers of mice with Atg7 deficient T cells contained fewer CD4^+^ and CD8^+^ T cells. Also in the hepatic T cell populations, the percentages of IFNγ and IL-17 secretion were increased but since the reduction in the percentages of CD4^+^ and CD8^+^ T cells is considerable, the total amount of IFNγ and IL-17 which is produced by T cells throughout the process of hepatic steatosis development was likely lower in Lck-Cre Atg7^f/f^ mice. It is unclear why the relatively few CD4^+^ and CD8^+^ T cells in the liver had a higher level of inflammatory cytokine secretion. Inhibition of autophagy in Th1 cells using 3-methyladenine or NH_4_Cl and leupeptin impairs IFNγ secretion (16), suggesting that it is unlikely that Atg7 deficiency increases Th1 differentiation resulting in enhanced IFNγ secretion. Interestingly, Treg cell-specific Atg7 deficiency has been described to induce a loss of FoxP3 and enhance their production of IFNγ and IL-17 (17). Likewise, knock out of Atg16L, another essential protein in autophagy, mimics the effects of Atg7 deficiency in Treg cells as Atg16L1 deficient Treg cells have increased IFNγ and IL-17 production (33). Though Atg7 deficiency in Treg cells impairs their survival and immunosuppressive capacity it also increases their homeostatic proliferation (17), suggesting that Atg7 deficient Treg cells are more resilient to defective autophagy as compared to Atg7 deficient conventional T cells. It is likely that the increase in IFNγ and IL-17 producing CD4^+^ T cells in the liver and spleen of Lck-Cre Atg7^f/f^ mice is partly due to Atg7 deficiency in Treg cells, which produce IFNγ and IL-17 under specific inflammatory conditions (17). In line, Treg cells with a Th1- or Th17-like phenotype have been described before in (models for) cardiovascular disease (34, 35). However, since barely any IFNγ producing CD4^+^ T cells were FoxP3^+^ in livers of normocholesterolemic Lck-Cre Atg7^f/f^ mice, the Treg cells which might have contributed to the increased percentage of IFNγ and IL-17 producing CD4^+^ T cells in the liver no longer express FoxP3 and are actually “former-Treg cells.” Another explanation for the increased percentage of IFNγ and IL-17 producing T cells which we observed in the liver is that Atg7 deficient Treg cells have impaired immunosuppressive capacity, which causes IFNy and IL-17 producing CD4^+^ T cells to be improperly inhibited by Treg cells. Since Treg cells represent a relatively small percentage of the total T cell population it is unlikely that the cells which caused the increased percentage of IFNy and IL-17 producing CD4^+^ T cells are exclusively (former) Treg cells. Further research is required to examine what the functional effects of autophagy deficiency in Th17 cells are and what their contribution to the observed T cell phenotype might have been.

Hepatic steatosis (or fatty liver) can develop when hepatocytes accumulate dietary lipids, potentially resulting in lipotoxicity. When this persists, immune cells such as Kupffer cells are activated and monocytes can be recruited when damaged or dead cells release danger signals such as damage-associated molecular patterns, leading to the development of non-alcoholic steatohepatitis (NASH) (25). *Ldlr*^−/−^ mice which are fed a high fat, high cholesterol diet are a suitable model to study the onset of inflammation in hepatic steatosis (32), suggesting the development of hepatic steatosis in virus-induced LDL receptor deficient mice is physiologically relevant. In the hepatic lymphocyte population, it is mainly Th17 cells which drive the development of steatosis (36). Through the secretion of IL-17, Th17 cells directly drive sinusoidal cells such as fat storing cells to produce type 1 collagen and activate macrophages to secrete inflammatory cytokines (37). Patients with hepatic steatosis have increased intrahepatic IL-17 expressing CD4^+^ T cells while in the blood, more IFNy secreting CD4^+^ T cells are detected as compared to healthy controls (36). In line, morbidly obese patients with NASH have higher intrahepatic gene expression of Th1-associated genes and a decreased ratio of IL-10/IFNy as compared to patients with non-alcoholic fatty liver disease (38). Whereas, Th17 and Th1 cells appear to drive NASH, Treg cells presumably inhibit its development as Treg cell deficient mice with WTD-induced atherosclerosis have more severe hypercholesterolemia due to impaired clearance of chylomicron remnants and very low density lipoproteins (39). Taken together, in our study we deem it most likely that the diminishment in the amount of hepatic T cells impaired the development of hepatic steatosis.

The contribution of NKT cells to the development of hepatic steatosis remains to be elucidated. In high-fat diet induced obesity, hepatic NKT cell numbers diminish, possibly contributing to the development of hepatic steatosis as the cytotoxicity-mediated killing of hepatocytes, which are under lipotoxicity-induced stress, is impaired (40, 41). The expansion of NKT cells during diet-induced steatosis development using probiotics actually protects against hepatic steatosis and insulin resistance (42). In contrast, expansion of hepatic NKT cells through the Hedgehog-pathway contributes to hepatic fibrosis (43), suggesting that the contribution of NKT cells to the pathogenesis of steatosis and NASH depends on the dynamics and inflammatory phenotype of hepatic NKT cells.

Studies describing the abundance of NKT cells in different organs during experimental atherosclerosis show contradictory results (44). Both an increase and a decrease in NKT cell number in atherosclerosis has been reported. In our experiments, the abundance of NKT cells was severely diminished in the livers of WTD-fed Lck-Cre Atg7^f/f^ mice, which is relevant as the liver contains the highest number of NKT cells in mice. This effect of Atg7 deficiency on hepatic NKT cell abundancy is due to reduced thymic NKT cell output as Atg7 deficiency inhibits the progression of NKT cells through the cell cycle and increases NKT cell apoptosis (45). Interestingly, Atg5 deficiency primarily hampers the secretion of IFNy by Th1-like NKT cells and Atg7 deficiency presumably has the same effect (45). Given their modulatory role in steatosis development, the lack of inflammation competent NKT cells diminished the development of hepatic steatosis. As LDLr^−/−^CD1d^−/−^ mice, which lack NKT cells, have similar cholesterol levels when fed a WTD as compared to LDLr^−/−^ mice (46) it is likely that in Lck-Cre Atg7^f/f^ mice, the combined effect of the lower numbers of inflammatory CD4^+^, CD8^+^, and of NKT cells inhibited the development of WTD-induced hepatic steatosis.

Atherosclerosis development was severely impaired in mice with T cell-specific Atg7 deficiency which can be explained by low levels of serum cholesterol and low numbers of CD4^+^ T cells, CD8^+^ T cells and NKT cells. Additionally, the lesions of Lck-Cre Atg7^f/f^ mice contained less collagen as compared to lesions from Atg7^f/f^ mice which is most likely due to more advanced and stabilized lesions in the latter group. The lack of a proper NKT cell population in Lck-Cre Atg7^f/f^ mice likely contributed to decreased lesion growth as NKT cells can drive atherogenesis through various mechanisms, including through perforin and granzyme-B mediated cytotoxicity and cytokine secretion (47–49). The fact that activated iNKT cells can decrease lesion stability by reducing collagen content (47) was likely overruled by their low abundancy in our study.

In this study we determined the effect of genetic blockade of autophagy in T cells on late stages of atherosclerosis in a mouse model where T cells lack functional autophagy from an early developmental stage and subsequently induced atherosclerosis. For a translation into a clinical setting in which cardiovascular patients could be treated with pharmacological autophagy inhibitors, it would be interesting to knock-out Atg7 using an inducible Cre mouse model in mice with pre-developed atherosclerosis. In addition it would have been highly interesting to dissect the effect of Atg7 deficiency in specific T cell subsets using mice with Cre recombinase under control of the promotor of T-bet, RORγt, and FoxP3 to induce Atg7 deficiency in Th1-, Th17-, and Treg cells, respectively.

In conclusion, T cell-specific Atg7 deficiency decreased the degree of diet-induced hepatic steatosis and atherosclerosis due to a decrease in numbers of CD4^+^, CD8^+^, and NKT cells. These results suggest that autophagy inhibition in T cells is feasible to diminish atherosclerosis. Further research focusing on the effect systemic administration of pharmaceuticals such as chloroquine has on non-T cells could contribute to its applicability to inhibit inflammation and potentially prevent cardiovascular disease.

## Author Contributions

JA and JK designed the experiments. JA, HD, FS, AF, MK, PvS, GvP, and IB performed the experiments. JA, IB, and JK wrote the manuscript.

### Conflict of Interest Statement

The authors declare that the research was conducted in the absence of any commercial or financial relationships that could be construed as a potential conflict of interest.
