# Supplementary material for: Defective Autophagy in T Cells Impairs the Development of Diet-Induced Hepatic Steatosis and Atherosclerosis
Source: Front Immunol. 2018 Dec 12;9:2937. doi: 10.3389/fimmu.2018.02937 (PMC6299070; doi:10.3389/fimmu.2018.02937)
Supplement: Supplementary file 1 [file Table_1.DOCX]

# Supplementary tables

| **Antigen** | **Label** | **Clone** | **Manufacterer** |
| --- | --- | --- | --- |
| fixable viability dye | eFluor 780 | n/a | ThermoFischer |
| Thy1.2 | PE-Cy7 | 53-2.1 | ThermoFischer |
| CD8 | FITC | 53-6.7 | ThermoFischer |
| IFNy | Alexa fluor 488 | XMG1.2 | ThermoFischer |
| IL-10 | APC | JES-16E3 | ThermoFischer |
| CD44 | APC | IM7 | ThermoFischer |
| CD44 | eFluor 450 | IM7 | ThermoFischer |
| CD62L | eFluor 450 | MEL-14 | ThermoFischer |
| CD62L | PerCP-Cy5.5 | MEL-14 | ThermoFischer |
| IL17 | PE | TC11-18H10 | BD Biosciences |
| CD4 | PerCP | RM4-5 | BD Biosciences |

Supplementary table 1 Antibodies used for flow cytometry

| Gene | Forward primer (5'-3') | Reverse primer (3'-5') |
| --- | --- | --- |
| *CD36* | atggtagagatggccttacttggg | agatgtagccagtgtatatgtaggctc |
| *Pparg* | aagccctttggtgactttatggagcc | tgcagcaggttgtcttggatgtcc |
| *Srebp2* | ccagctcctgggtgagacctac | caggcgcacagtggcttcat |
| *Scd1* | ggaaagtgaggcgagcaactgacta | caggacggatgtcttcttccaggtg |
| *Fas* | gctgttttcccttgctgcagacatg | aacccgcctcctcagctttaaactc |
| *Il10* | gggtgagaagctgaagaccctc | tggccttgtagacaccttggtc |
| *CD68* | tgcctgacaagggacacttcggg | gcgggtgatgcagaaggcgatg |
| *Infg* | ccttcttcagcaacagcaaggcga | gcgctggacctgtgggttgt |
| *Il17* | tcatgtggtggtccagctttccctc | actacctcaaccgttccacgtcacc |
| *Fdft1* | aacatgcctgccgtcaaagctatca | gcttgatgatgggtctgagttgggg |
| *Acaa2* | cttgaccccagcaaaaccaatgtgag | gatcccactgcgtactttccacctc |
| *Mcp1* | ctgaagccagctctctcttcctc | ggtgaatgagtagcagcaggtga |
| *Eef2* | gaacaggaagcgtggccatgtgttt | ggctgctgttgtcaaaaggatcccc |
| *36B4* | ctgagtacaccttcccacttactga | cgactcttcctttgcttcagcttt |

Supplementary table 2 List of primers used for qPCR expression analysis. Expression of genes were normalized to housekeeping genes *Eef2* and *36B4*.
