# Supplementary figures and images for: Defective Autophagy in T Cells Impairs the Development of Diet-Induced Hepatic Steatosis and Atherosclerosis
Source: Front Immunol. 2018 Dec 12;9:2937. doi: 10.3389/fimmu.2018.02937 (PMC6299070; doi:10.3389/fimmu.2018.02937)

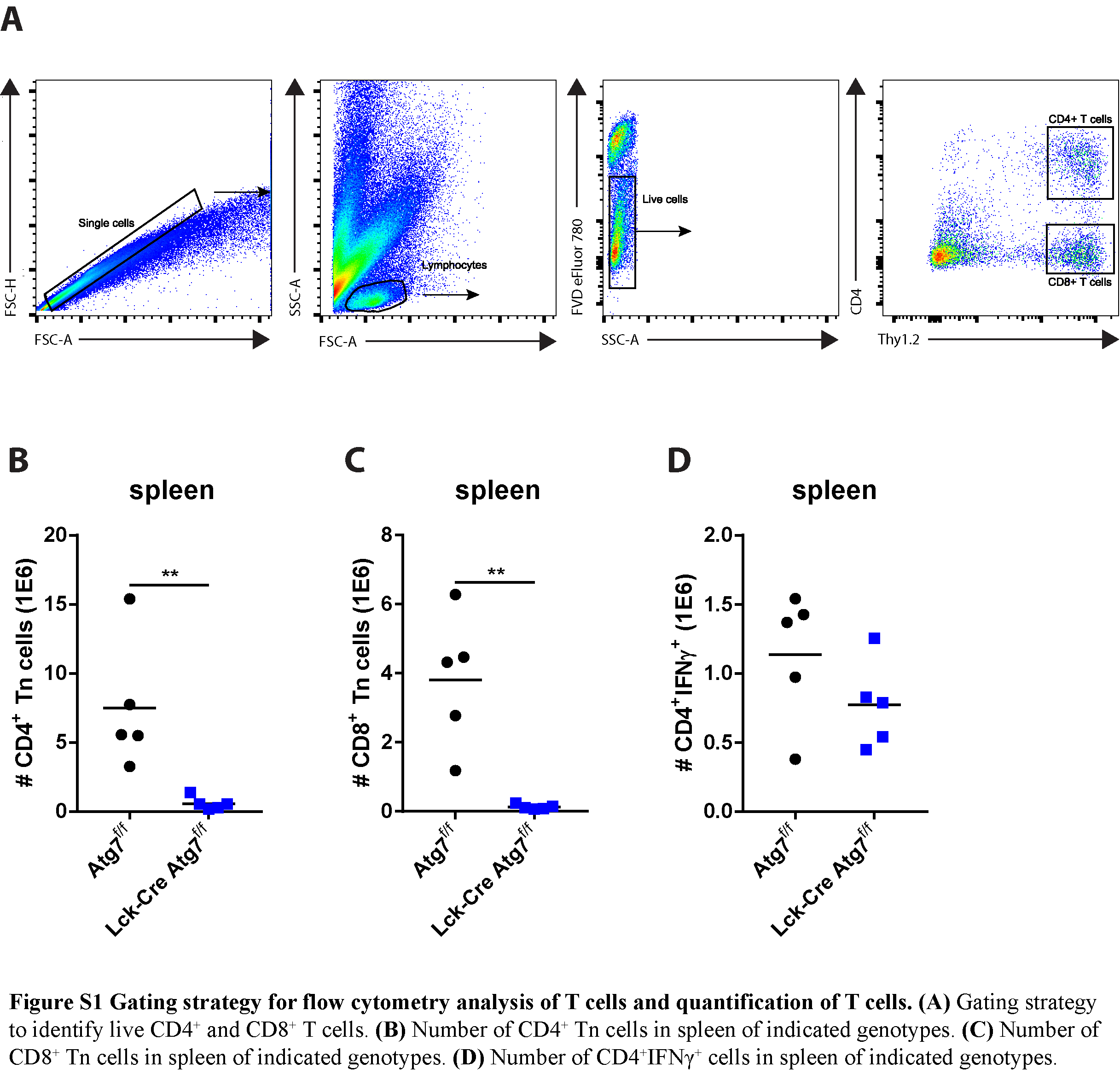

Supplement: Supplementary file 2 [file Image_1.TIF]

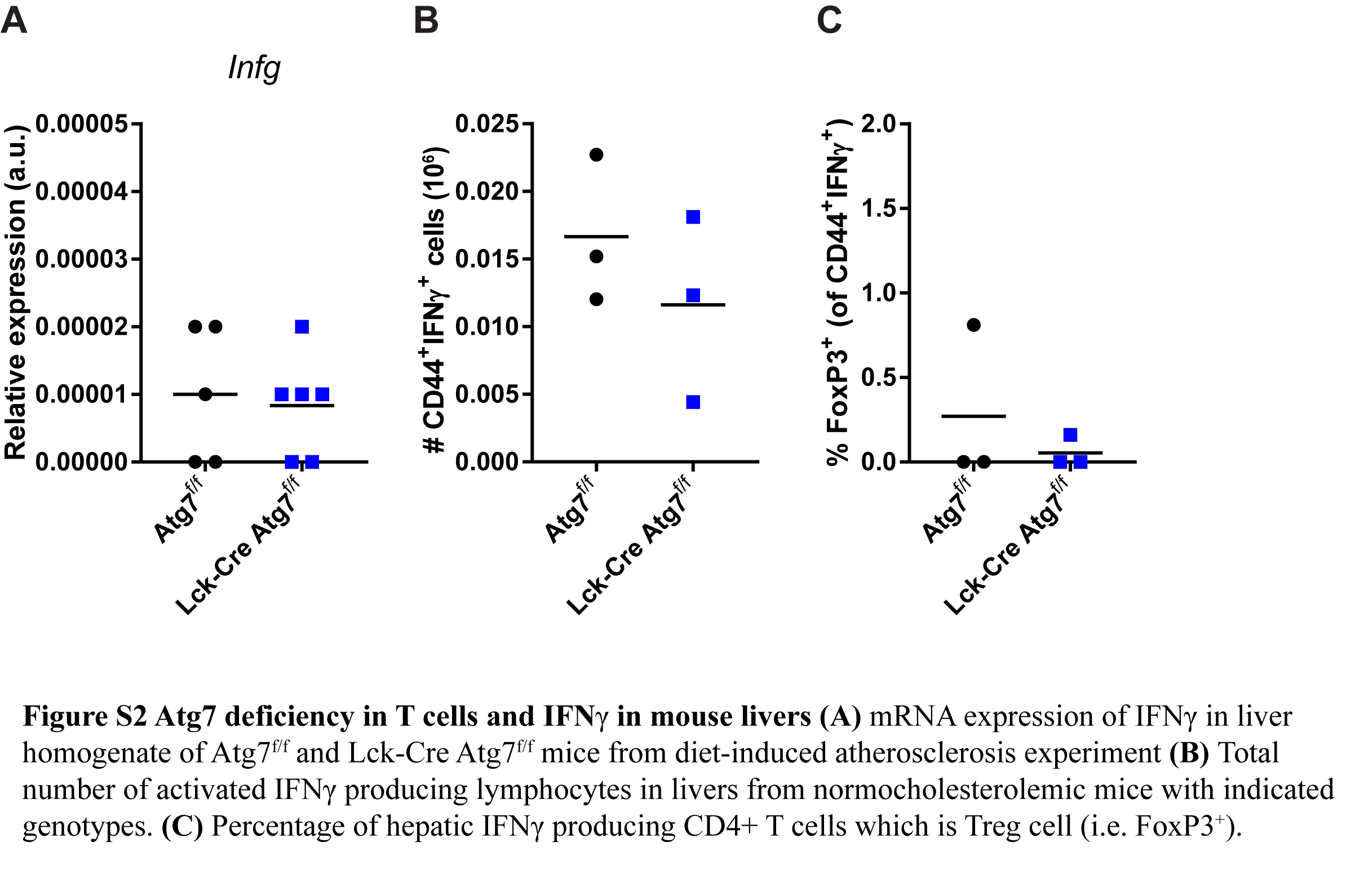

Supplement: Supplementary file 3 [file Image_2.TIF]

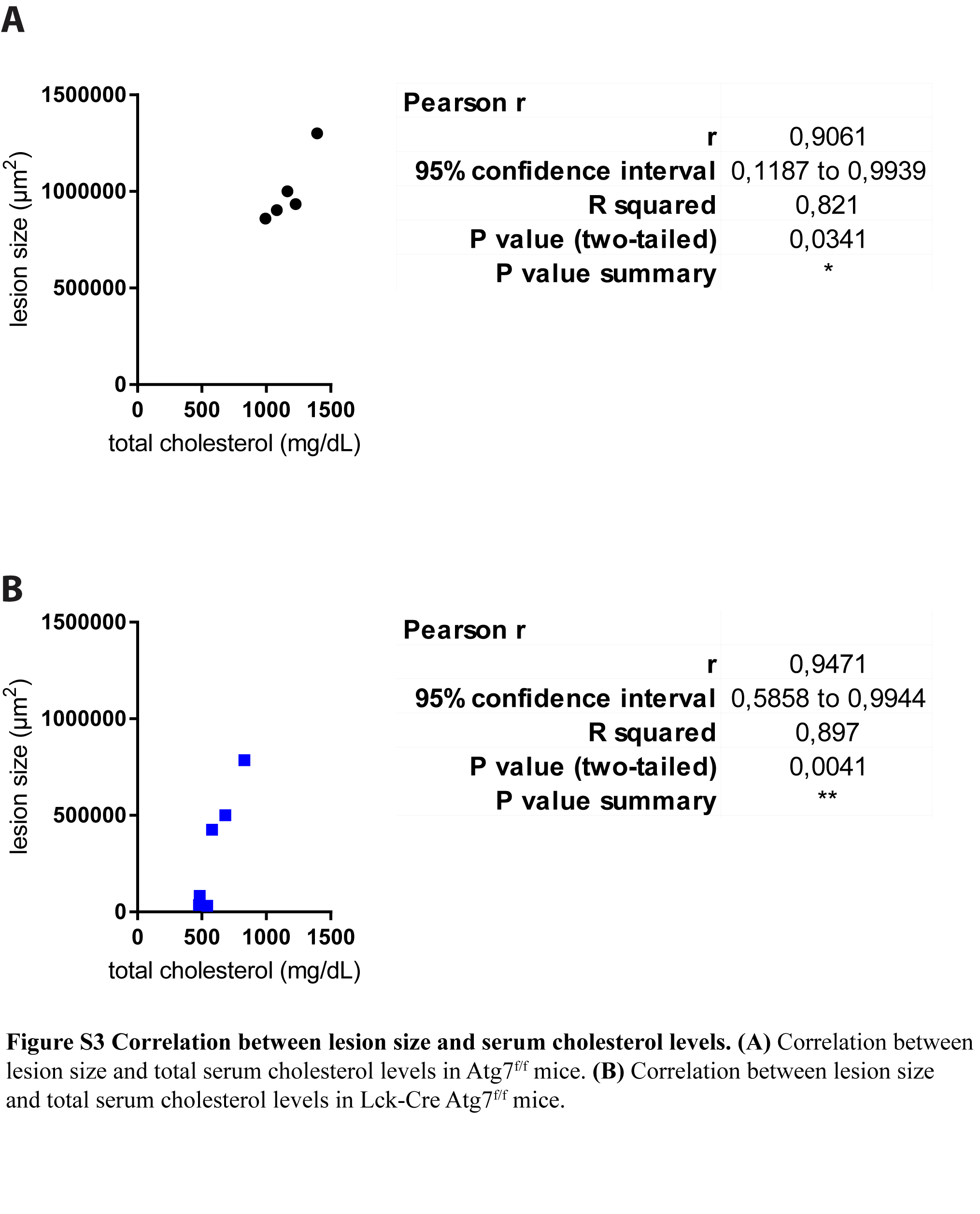

Supplement: Supplementary file 4 [file Image_3.TIF]
